# Structural variation analysis of 6,500 whole genome sequences in amyotrophic lateral sclerosis

**DOI:** 10.1038/s41525-021-00267-9

**Published:** 2022-01-28

**Authors:** Ahmad Al Khleifat, Alfredo Iacoangeli, Joke J. F. A. van Vugt, Harry Bowles, Matthieu Moisse, Ramona A. J. Zwamborn, Rick A. A. van der Spek, Aleksey Shatunov, Johnathan Cooper-Knock, Simon Topp, Ross Byrne, Cinzia Gellera, Victoria López, Ashley R. Jones, Sarah Opie-Martin, Atay Vural, Yolanda Campos, Wouter van Rheenen, Brendan Kenna, Kristel R. Van Eijk, Kevin Kenna, Markus Weber, Bradley Smith, Isabella Fogh, Vincenzo Silani, Karen E. Morrison, Richard Dobson, Michael A. van Es, Russell L. McLaughlin, Patrick Vourc’h, Adriano Chio, Philippe Corcia, Mamede de Carvalho, Marc Gotkine, Monica P. Panades, Jesus S. Mora, Pamela J. Shaw, John E. Landers, Jonathan D. Glass, Christopher E. Shaw, Nazli Basak, Orla Hardiman, Wim Robberecht, Philip Van Damme, Leonard H. van den Berg, Jan H. Veldink, Ammar Al-Chalabi

**Affiliations:** 1 King’s College London, Maurice Wohl Clinical Neuroscience Institute, Department of Basic and Clinical Neuroscience, De Crespigny Park, London, UK; 2 Department of Biostatistics and Health Informatics, Institute of Psychiatry, Psychology and Neuroscience, King’s College London, London, UK; 3 Department of Neurology, UMC Utrecht Brain Center, Utrecht University, Utrecht, The Netherlands; 4 KU Leuven – University of Leuven, Department of Neurosciences, Experimental Neurology; VIB Center for Brain & Disease Research, Laboratory of Neurobiology, Leuven, Belgium; 5 Sheffield Institute for Translational Neuroscience (SITraN), University of Sheffield, Sheffield, UK; 6 Complex Trait Genomics Laboratory, Smurfit Institute of Genetics, Trinity College Dublin, Dublin, Ireland; 7 Department of Neurology and Laboratory of Neuroscience, IRCCS Istituto Auxologico Italiano and Department of Pathophysiology and Transplantation, “Dino Ferrari” Center, Università degli Studi di Milano, Milano, Italy; 8 Koc University, School of Medicine, Translational Medicine Research Center- NDAL, Istanbul, Turkey; 9 Mitochondrial pathology Unit, Instituto de Salud Carlos III, Madrid, Spain; 10 Neuromuscular Diseases Unit/ALS Clinic, Kantonsspital St. Gallen, St. Gallen, Switzerland; 11 Faculty of Medicine, Health and Life Sciences, Queen’s University Belfast, Belfast, Northern Ireland UK; 12 Institute of Health Informatics, University College London, London, UK; 13 Centre SLA, CHRU de Tours, Tours, France; 14 Rita Levi Montalcini, Department of Neuroscience, ALS Centre, University of Torino, Turin, Italy; 15 Azienda Ospedaliera Citta della Salute e della Scienza, Torino, Italy; 16 Federation des Centres SLA Tours and Limoges, LITORALS, Tours, France; 17 Physiology Institute, Faculty of Medicine, Instituto de Medicina Molecular, University of Lisbon, Lisbon, Portugal; 18 Hadassah University Hospital, Jerusalem, Israel; 19 Neurology Department, Hospital Universitari de Bellvitge, Barcelona, Spain; 20 Hospital San Rafael, Madrid, Spain; 21 Department of Neurology, University of Massachusetts Medical School, Worcester, MA USA; 22 Department of Neurology, Center for Neurodegenerative Diseases, Emory University, Atlanta, GA USA; 23 King’s College Hospital, Denmark Hill, London, UK; 24 Academic Unit of Neurology, Trinity College Dublin, Trinity Biomedical Sciences Institute, Dublin, Republic of Ireland; 25 Department of Neurology, Beaumont Hospital, Dublin, Republic of Ireland; 26 Neurology Department, University Hospitals Leuven, Leuven, Belgium

**Keywords:** Amyotrophic lateral sclerosis, Comparative genomics

## Abstract

There is a strong genetic contribution to Amyotrophic lateral sclerosis (ALS) risk, with heritability estimates of up to 60%. Both Mendelian and small effect variants have been identified, but in common with other conditions, such variants only explain a little of the heritability. Genomic structural variation might account for some of this otherwise unexplained heritability. We therefore investigated association between structural variation in a set of 25 ALS genes, and ALS risk and phenotype. As expected, the repeat expansion in the *C9orf72* gene was identified as associated with ALS. Two other ALS-associated structural variants were identified: inversion in the *VCP* gene and insertion in the *ERBB4* gene. All three variants were associated both with increased risk of ALS and specific phenotypic patterns of disease expression. More than 70% of people with respiratory onset ALS harboured *ERBB4* insertion compared with 25% of the general population, suggesting respiratory onset ALS may be a distinct genetic subtype.

## Introduction

Amyotrophic lateral sclerosis (ALS) is a neurodegenerative disease predominantly of motor neurons, characterized by progressive weakness of the limbs, trunk, diaphragm, and bulbar musculature, with death occurring from respiratory failure, typically within 3 years of onset. Despite the poor prognosis, there is considerable variation in the survival rate, and up to 10% of people with ALS live more than 8 years from first symptoms^1^. In about 25% of people, the first symptom is difficulty with speaking or swallowing, and in nearly all the rest, it is limb weakness. However, about 1% to 2% of people experience onset with diaphragmatic weakness and early respiratory failure^2,3^. No gene variant has been found to predispose to a specific site of onset without also predisposing to greater risk of ALS. For example, pathological hexanucleotide expansion in the *C9orf72* gene, a cause of ALS, increases the risk of bulbar onset^4^. The possibility that respiratory onset ALS represents a distinct subgroup is supported by the observation that despite early diaphragm involvement, disease progression is in some cases surprisingly slow^5^.

Genome-wide association studies have identified ALS risk variants that are relatively common in the population, but such alleles tend to have small effect sizes and can explain only a small proportion of heritability^6,7^. The remaining heritability is presumed to lie in other genomic variation, including rare variants, repeat sequences and structural variants, not easily tagged by SNPs.

Structural variants comprise various forms of genomic imbalance such as insertions, deletions, inversions, duplications and inter-chromosomal translocations^8^. Such variants have been associated with various neurological and psychiatric diseases including Charcot-Marie-Tooth neuropathy^9^, schizophrenia^10^ and autism^11,12^. Attempts to understand the relationship of structural variation with ALS have been limited by sequencing technology, computational burden, and the small number of samples^13,14^. Measuring the intensity of signals derived from a genotyping array is the most used method in detecting copy number variants^15,16^, but advances in sequencing technology and increased computing power have now made it feasible to study structural variation by more direct means^17^.

Here, we report the analysis of structural variation in known ALS genes using 6,580 whole genome sequences and genotype-phenotype correlations, using the Project MinE whole genome sequencing and deep phenotype dataset^18^.

## Results

### Sample characteristics

There were 6,580 whole genome sequences, reducing to 6,195 samples (4,315 from people with ALS and 1,880 controls) after quality control, with minimum ~25× coverage across each sample. Of those with ALS, 4,236 had apparently sporadic ALS and 79 had familial ALS. The male-female ratio was 2:1. Overall, 31 had cognitive impairment, 20 had ALS-frontotemporal dementia (ALS-FTD) and 63 had respiratory onset ALS. There were 4,287 people sequenced using the HiSeqX Illumina platform, and 1,908 sequenced using the HiSeq2000 platform (Table 1).

**Table 1:** Demographic features of the study population.

Cohort	Sample	Case	Control	Female	Male
Belgium	548	368	180	209	339
Ireland	403	267	136	161	242
Netherlands	2894	1859	1035	1182	1712
Spain	338	233	105	145	193
Turkey	223	148	75	87	136
United Kingdom	1402	1124	278	603	799
United States	387	316	71	153	234
Total	6195	4315	1880	2540	3655

Detailed demographic features of the study population.

### Association analyses

In three of the 25 genes, structural variation was associated with ALS: *C9orf72* gene hexanucleotide repeat expansion (odds ratio 28.1, 95% CI (10.45, 75.61), *p* = 2 × 10^−16^), inversion in the *VCP* gene (odds ratio 2.33, 95% CI (2.09, 2.61), *p* = 2 × 10^−5^) and insertion in the *ERBB4* gene (odds ratio 2.55, 95% CI (2.26, 2.88), *p* = 3 × 10^−5^; (Table 2, Supplementary appendix Table 2–6). All passed the multiple testing correction threshold (*p* = 0.0005). Inspection of the sequences showed that there were no rare missense or loss of function variants in those with *ERBB4* insertion. In two people (0.1%) with *VCP* inversion, such variants were found. Inspection of BAM files showed that structural variation calls in the VCF files had a corresponding appropriate change in the BAM file. Inversions and insertions were not identical between people and the *p*-value (*p* = 2 × 10^−16^) is the minimum *p*-value that R will report to the console.

**Table 2:** Structural variation in sporadic ALS.

Gene	p-value	SV-type	Cases (freq)	Controls (freq)	Odds ratio (CI 95%)
*C9orf72*	2 × 10^−16^	Expansion	244 (0.06)	4 (0.002)	28.1 (10.45, 75.61)
*VCP*	2 × 10^−5^	Inversion	2430 (0.56)	669 (0.36)	2.33 (2.09, 2.61)
*ERBB4*	3 × 10^−5^	Insertion	2001 (0.46)	476 (0.25)	2.55 (2.26, 2.88)

There were three genes in which structural variation was associated with ALS: *C9orf72*, *VCP*, and *ERBB4*. Odds ratio is calculated from the exponential of the beta from the regression model including principal components of ancestry and other confounders. *SV* structural variation, *freq* frequency.

In the 200 samples that we tested for validation, the *VCP* inversion was detected by Manta alone in 180 samples, Pindel alone in 170, both Manta and Pindel in 165 and by neither in 35. The *ERBB4* insertion was detected by Manta alone in 120 samples, Pindel alone in 130, both Manta and Pindel in 113, and by neither in 87. Comparison of Manta 0.23.1 results and the more recent version of Manta, 1.6.0, showed no difference in the number of samples showing inversion in the *VCP* gene identified by either version. The same was true for insertion in the *ERBB4* gene.

### Age of onset and age of death analyses

The mean age of onset for all people with apparently sporadic ALS was 60.7 years (SD 11.84) and the mean age at death was 65.3 years (SD 10.61). The Kolmogorov-Smirnov test showed non-normal distributions for both datasets (*p* < 0.001). The test for skewness showed −0.33 for age of onset and −0.48 for age of death, indicating an approximately symmetric distribution.

The mean age of onset in people with *C9orf72* gene expansion was 2.7 years younger than those with no *C9orf72* gene expansion (*p* = 8.8 × 10^−8^, 95% CI for the difference 1.2 to 4.2 years). The mean age of onset in people with *VCP* gene inversion was 3 years younger than for people with no *VCP* gene inversion (*p* = 4.2 × 10^−13^, 95% CI for the difference 2.2 to 3.7 years). Additionally, the mean age of onset in those with *ERBB4* gene insertion was one year younger than for those with no *ERBB4* insertion (*p* = 0.003, 95% CI for the difference 0.25 to 1.72 years). The mean age of onset in people with *VCP* inversion, *ERBB4* insertion and *C9orf72* gene expansion was 3.5 years younger than those with no with no reported structural variation in these genes (p = 0.001, 95% CI for the difference 1.3 to 5.6 years) (Table 3).

**Table 3:** Structural variation burden for age of onset.

	SV absent	SV present		
Gene	Age of onset (years)	Age of onset (years)	*p*-value	Difference in years
*C9orf72*	62.0	58.8	8.8 × 10^−8^	3.2 (4.31-1.96)
*VCP*	62.6	59.7	4.2 × 10^−13^	2.97 (2.22-3.72)
*ERBB4*	61.2	60.2	0.003	1.00 (0.25-1.72)
*combined group*	62.6	59.3	0.001	3.5 (1.3 −5.6)

*SV* structural variation.

People with ALS and *C9orf72* gene expansion died on average 3.8 years younger than people with ALS and no *C9orf72* gene expansion (*p* = 2.3 × 10^−9^ 95% CI for the difference 2.6 to 5.1 years). People with ALS and *VCP* gene inversion died on average 1.8 years younger than those with ALS and no *VCP* gene inversion (*p* = 1.4 × 10^−5^, 95% CI for the difference 1.0 to 2.5 years). No difference in age at death was observed between people with ALS and *ERBB4* gene insertion and those with ALS and no *ERBB4* gene insertion (*p* = 0.1). People with ALS and *VCP* inversion, *ERBB4* insertion and *C9orf72* gene expansion died on average 4.8 years younger than those with no reported structural variations in those genes (*p* = 5.0 × 10^−4^, 95% CI for the difference 1.9 to 6.7 years) (Table 4).

**Table 4:** Structural variation burden for age of death.

	SV absent	SV present		
Gene	Age of death (years)	Age of death (years)	p-value	Difference in years
*C9orf72*	66.0	62.2	2.3 × 10^−9^	3.8 (2.64−5.10)
*VCP*	66.7	64.8	1.4 × 10^−5^	1.8 (1.04−2.58)
*ERBB4*	65.9	65.0	0.1	NA
*combined group*	66.5	62.6	5.0×10^−4^	4.8 (1.9−6.7)

*SV* structural variation.

A family history of ALS was associated with a younger age at onset (4 years, *p* = 0.02, 95% CI for the difference 0.37 to 5.96 years) and death (4.5 years, p = 0.01, 95% CI for the difference 1.1 to 7.8 years), when compared with those with no family history. However, no difference in age of onset or death was observed when those with a family history were compared against those with no family history and carrying structural variation in the *C9orf72, VCP* or *ERBB4* genes, suggesting these genetic variations are themselves reducing the age of onset and death.

### Survival analyses

Cox survival analysis showed that people with ALS and *C9orf72* gene expansion had worse survival (*p* = 3.0 × 10^−6^) than people with ALS with no *C9orf72* gene expansion (Supplementary Fig. 2), while people with ALS and *VCP* gene inversion had longer survival than those with ALS and no *VCP* gene inversion (*p* = 0.002, Supplementary Fig. 3). No difference in survival was observed between people with ALS and *ERBB4* gene insertion and those with ALS and no *ERBB4* gene insertion (*p* = 0.9) (Supplementary Fig. 4). People with *C9orf72* gene expansion, *VCP* gene inversion, and *ERBB4* gene insertion had worse survival (*p* = 6.7 × 10^−5^) than people with ALS with no overlapping structural variation in *C9orf72*, *VCP*, and *ERBB4* genes (Supplementary Fig. 7).

### Site of onset analyses

Multivariable linear regression showed an association between *C9orf72* repeat expansion and bulbar site of onset (*p* = 0.01), confirming previous findings. Inversion in the *VCP* gene was associated with bulbar onset (*p* = 3.5 × 10^−12^) and frontotemporal dementia (*p* = 1.1 × 10^−4^). *ERBB4* insertion increased the risk of ALS, and also increased the risk of respiratory onset. *ERBB4* insertion was seen in 45 of the 63 people (71.4%) with respiratory onset ALS (Table 5). The odds ratio of respiratory compared with non-respiratory onset was 2.9 (95% CI 1.69-5.08; *p* = 6.2 × 10^−5^), but compared with controls, the odds ratio was 7.37 (95% CI 4.23, 12.86; *p* = 4.4 × 10^−16^). Kaplan–Meier survival analysis showed that people with ALS with respiratory onset had worse survival than those with spinal onset ALS, and better survival than those with bulbar onset ALS (log rank *p* = 6.6 × 10^−34^) (Supplementary Fig. 5) but in the subset with *ERBB4* insertion there was no difference in survival (log rank *p* = 0.15) (Supplementary Fig. 6). *ERBB4* insertion was seen in 20 of the 31 with cognitive impairment OR 2.3 CI 95% (1.09–4.98; *p* = 1.3 × 10^−4^). We could not determine whether the cognitive changes were a result of respiratory failure, frontotemporal impairment, or some other cause. Moreover, individuals who harboured multiple types of structural variation were more likely to develop FTD and cognitive changes (*p* = 0.001), but none had respiratory onset ALS.

**Table 5:** *ERBB4* insertion in respiratory onset ALS.

ERBB4 insertion	Respiratory onset (freq)	Non-respiratory onset (freq)	Controls (freq)
Present	45 (0.71)	1956 (0.46)	476 (0.25)
Absent	18 (0.29)	2296 (0.53)	1404 (0.75)
Total	63	4252	1880

*ERBB4* insertion in respiratory onset ALS compared with non-respiratory onset ALS and controls. *Freq* frequency.

## Discussion

We have shown that genomic structural variants in the *C9orf72, VCP*, and *ERBB4* genes are variously associated with ALS risk, younger age of onset, earlier age at death, specific sites of onset, and survival, highlighting the importance of structural variation events in ALS.

Earlier studies, using smaller sample sizes and attempting to impute structural variation from SNP microarray data, found no evidence of a difference in global structural variation burden between ALS and controls^13,14,19^. Our study has the advantage of directly sequenced data, giving a high degree of confidence for structural variant calling, and a larger sample size, giving a higher degree of confidence for statistical analyses, although even larger studies would be ideal. As calling structural variants is dependent on the quality of sequencing data, we applied stringent quality control measures, excluding 385 samples. The final number of samples passing quality control was 6195 whole genome sequences each representing one individual, and making this one of the largest such datasets in the world.

In keeping with previous findings, we have found genotype-phenotype correlations in risk genes for ALS, the most striking of which is the finding of insertion in the *ERBB4* gene in 71.4% of people with respiratory onset ALS compared with 46.4% of those with non-respiratory onset, and just 25.3% of the general population. This is the largest genetic study of respiratory onset ALS, but because the frequency of respiratory onset in ALS is only about 1 to 2%^2,3,20^, the absolute numbers are still small. Nevertheless, the finding is possible because such a large proportion of affected people have the same genetic variation. The odds ratio of more than seven means this is a moderately large effect, and much larger than is typically seen in association studies. Interestingly, the original *ERBB4* report has a pedigree in which affected individuals had a similar mean age of onset and with one in five also having respiratory onset^21^. We also found that insertion in the *ERBB4* gene was associated with cognitive change. Multiple previous studies have linked *ERBB4* gene variation with FTD and cognitive or behavioural changes^22–26^.

As expected, we confirmed the worse prognosis conferred by *C9orf72* expansion mutation, but other phenotypic associations of *C9orf72* are less well understood. Previous studies of the *C9orf72* repeat expansion and onset age have led to conflicting results^27–30^, and the correlation between repeat size and diagnosis is poorly understood in apparently sporadic ALS, as most studies have been in familial ALS^31–33^. We found that familial ALS is associated with a younger age of onset, consistent with previous studies, and that this is also true for those with *C9orf72* expansion mutation, regardless of family history^34–36^. Furthermore, our results support previous studies finding that the frequency of the *C9orf72* expansion mutation in the general population is about 0.2%^37^. Previous independent research has shown *C9orf72* repeat expansion in healthy individuals in whom the expansion was confirmed by standard laboratory methods^37^. To confirm that the expansion can be seen in unaffected individuals, we have calculated the number of controls with *C9orf72* repeat expansion in new data in the Project MinE dataset and found 10 more with this expansion. While it might seem strange that a major ALS risk gene should be seen in unaffected control individuals, it is in fact expected, since there is age-dependent penetrance, penetrance is incomplete, and the effects of the expansion mutation are pleiotropic, increasing risk for several conditions other than ALS. The rate we observe is similar to that seen in other studies and in public databases.

*VCP* inversion is associated with longer survival as well as younger age of onset. Our findings also suggest that *VCP* structural variation might be a marker for cognitive impairment and ALS-FTD, supporting previous work showing an association of common variation in the *VCP* gene with FTD and cognitive impairment^26,38,39^.

Although, age of onset can be a good predictor of disease course, age of onset is not determinative of age at which death occurs. We have shown that both age of onset, age at death and disease duration are highly variable between individuals and genetically influenced. The genetic associations we have found in apparently sporadic ALS are in genes previously identified from family-based studies (*C9orf72, ERBB4* and *VCP*) supporting the notion that familial and sporadic ALS are not mutually exclusive categories but rather a spectrum^36,40–42^. Understanding the involvement of SVs in *VCP* and *ERBB4* therefore might help in understanding disease trajectories in ALS and potentially therefore selection in clinical trials. Moreover, understanding trajectories of illness is useful for planning clinical care.

Interestingly, those who harboured multiple types of structural variation were found to have a younger age of onset, younger age of death and worse survival than people with for example, *C9orf72* expansion alone, implying that people with multiple mutations of large effect in ALS driver genes might need fewer than six molecular steps to develop ALS^40,41^. Given the relative frequencies of the variants, screening for *VCP* inversion in people with *C9orf72* expansion might therefore be helpful in estimating prognosis.

This study has several limitations. We have analysed whole genome sequencing data generated using two sequencing platforms, the HiSeqX Illumina platform, and the HiSeq2000 platform, which increases the possibility of a batch effect. However, cases and controls are similarly distributed between the platforms (HiSeq2500 66% cases, 34% controls and HiSeqX 70% cases and 30% controls) (*p* = 0.54). To overcome this potential weakness, all the samples used were sequenced at the same Illumina lab using two industry-leading sequencing platform for all samples, as well as designing the study to minimize batch effects by having cases and controls sharing the same sequencing plate, and taking sequencing platform into account as a covariate in our analyses. Furthermore, although we have assessed reported ALS-associated rare missense and loss of function variants in linkage disequilibrium with the structural variants, we cannot exclude the possibility that the differential risk and phenotypes observed could be modulated by small common single-nucleotide variants or indels in linkage disequilibrium with the structural variants. Using GeneVar SV data browser^43^, the estimated frequency of *VCP* inversion is 0.0005 and *ERBB4* insertion 0.82. However, allele frequencies tend to vary across human populations, and different SV callers may give varying results between datasets. To allow full comparison between studies therefore requires the sequencing platform, population tested and SV callers are identical^44^. Another limitation is that we restricted the analysis of structural variation to known ALS genes. Extending the analysis to the entire genome would give a comprehensive view of this type of genomic variation in ALS, but with current technology is extremely resource intensive. Finally, ALS is a disease of the central nervous system, but our WGS data are derived from leukocyte DNA, since our DNA source was whole blood, and somatic mutation affecting the nervous system cannot therefore be assayed with our method. However, our findings have the advantage of a large sample size of more than 4300 cases.

Our analysis was restricted to a statistical genomics approach. Although the sample size is large, replicating our results in other ALS datasets and performing wet lab confirmation will be needed to validate the findings. We are reassured by the observation that VCF calls were matched with raw BAM file reads when tested.

Analysis of structural variation shows that such genetic variations influencing site of onset also modify risk, as is true for single nucleotide variations. Our finding that 71.4% of people with respiratory onset ALS have insertion in the *ERBB4* gene is an important clue to disease mechanism and factors that determine which group of motor neurons are most vulnerable at disease onset, a key issue in neurodegenerative disease research. Although the number of people with ALS with respiratory onset in our study is small compared with that for other phenotypes, this is the largest genetic study of respiratory onset ALS thus far. The finding of association is possible because of the homogeneity of the cause, corresponding to a large effect of the genetic variation identified.

In this large study of structural variation in ALS using whole genome sequence data, we find a number of risk variants for ALS as well as structural variants corresponding to specific ALS phenotypes. Further work is needed to understand the mechanisms and pathways underlying these relationships.

## Methods

### Data sources

Samples were from the international Project MinE whole genome sequencing consortium and derived from seven countries: the USA, Ireland, Belgium, the Netherlands, Spain, Turkey, and the United Kingdom^18^.

### Ethical approval

Informed consent for genetic research was obtained from all participants, approved by the Trent Research Ethics Committee 08/H0405/60.

#### Phenotyping

Clinical information including sex, age at first symptoms, age at onset, site of onset, survival status, and disease duration, was obtained from the patient record according to standard definitions as defined by the SOPHIA standard operating procedures^45^.

### Whole-genome sequencing

DNA was isolated from venous blood using standard methods. DNA concentration was set at 100 ng/µl as measured by fluorimeter with the PicoGreen® dsDNA quantitation assay. DNA integrity was assessed using gel electrophoresis. All samples were sequenced using Illumina’s FastTrack services (San Diego, CA, USA) on the Illumina HiSeq 2000 (100 bp paired-end reads) and HiSeqX platforms (150 bp paired end reads)^46^, using PCR-free library preparations. Binary sequence alignment/map formats (BAM) were generated for each individual. The Project MinE genomes were aligned with Isaac (Illumina) to hg19. The details of the Isaac alignment and variant calling pipelines are discussed in Project MinE design^18^ and Isaac protocol^47^.

### Determination of pathogenic ALS gene variants

A panel of 25 ALS genes was tested (*ALS2*, *ANG*, *ATXN2*, *C9orf72*, *CHCHD10*, *DAO*, *ERBB4*, *FUS*, *HNRNPA1*, *MOBP*, *NEK1*, *OPTN*, *PFN1*, *SCFD1*, *SETX*, *SOD1*, *SPG11*, *SQSTM1*, *TARDBP*, *TBK1*, *TUBA4A*, *UBQLN2*, *UNC13A*, *VAPB*, and *VCP*)^1,22,48^ (Table 1. Supplementary appendix) selected for harbouring large-effect, rare, Mendelian ALS gene variants or common variants showing well-replicated association. The *SMN1* gene is being assessed independently within the Project MinE consortium and was therefore not included in this study.

Manta V 0.23.1^49^ was used for variant assembly, variant extraction, and genomic quality scoring. A VCF was then generated for each participant. As the calls for Manta in this study were done using version 0.23.1, we repeated the test in a subset of 100 samples using the most recent version of Manta V1.6.0. For validation of Manta 0.23.1 calls, we tested the main SVs using a second tool, Pindel, in 200 randomly selected samples.

To calculate the number of structural variation types in each gene, an in-house pipeline was used to filter the variants according to quality score, size, and type of structural variant. Insertions with size less than 200 bp were excluded as recommended by the Manta protocol.

Repeat primed PCR or Expansion Hunter-v2.5.1^50^ were used to assay the hexanucleotide repeat expansion in the *C9orf72* gene.

In individuals with structural variation, sequences were inspected for rare missense or loss of function variants known to be associated with ALS to exclude linkage disequilibrium of the structural variant with the rare variant as an explanation of association.

### Statistical analysis

The effect of structural variation on ALS risk in each gene was examined independently, assessed using multivariable linear regression after correcting for different sequencing platforms and population stratification, principal components, centre, age and sex. To test gene-gene interaction effects between the identified structural variation groups, a combined group was created for any type of structural variation to compare against individuals with no structural variation in the genes examined.

For age of onset data and age of death, a test of normality was conducted using the Kolmogorov-Smirnov test of normality. Skewness values were also obtained.

As the age of onset and the age of death were not normally distributed, the median age of onset and age of death between people with sporadic ALS with structural variation, people with sporadic ALS without structural variation, and people with familial ALS, was compared with the non-parametric Mann-Whitney *U* test with 0.95 confidence level. To estimate the size of any ascertainment bias observed, the median time between symptom onset and diagnosis was compared between those with familial ALS and those with apparently sporadic ALS in a Mann-Whitney *U* test.

Genotype-phenotype association for site of symptom onset (bulbar muscles, limb, respiratory) and presence of cognitive impairment for each gene was examined independently, assessed using multivariable linear regression after correcting for different sequencing platforms and population stratification, principal components, centre, age and sex.

To assess the effect of structural variation on survival, we used Cox regression, controlling for age of onset, sex, *C9orf72* expansion status, principal components, centre and technology platform and site of disease onset (bulbar muscles, limb or respiratory, supplementary appendix Table 7). To assess survival in respiratory onset ALS we also used Kaplan-Meier survival analysis.

Statistical tests were performed using IBM SPSS Statistics 24.0 (SPSS Inc., Illinois), RStudio, R Foundation for Statistical Computing 3.4.1.

We tested four structural variation categories: deletion, insertion, inversion, and duplication, in 25 genes. Therefore, we used 0.0005 [0.05/(25*4)] as the Bonferroni-corrected threshold for multiple testing correction.

### Quality control

There were 6,195 samples (4,315 from people with ALS and 1,880 controls) passing quality control from a total of 6580 whole genome sequences. Quality control was preformed separately on genotyped data of each population according to the Project MinE methods published previously^51^. Sample mismatch was tested using sex checks, where genetic sex was compared to reported gender. After quality control, the full set of genomic Variant Call Format files (gVCFs) were merged together by first converting the gVCFs to Plink format and then merging all files together. This generated a single dataset containing all variant sites across all individuals. Non-autosomal chromosome and multi-allelic variants were excluded from pilot analyses. Sample and SNP quality control were performed using Plink^51,52^ and VCFtools^53^. To begin sample quality control, missingness by sample was calculated on a per-chromosome basis.

All other sample quality control steps were performed on a set of high-quality biallelic SNPs that had minor allele frequency at least 10%, missingness < 0.1%, were linkage disequilibrium pruned at an *r*^2^ threshold of 0.2, were not A/T or C/G SNPs, did not lie in the major histocompatibility complex or lactase gene locus, and did not occur in the inversions on chromosome 8 or chromosome 17. The ~30,000 SNPs overlapping this set of SNPs and HapMap 3 were used to calculate principal components projecting the ALS cases and controls onto the HapMap 3 samples. Samples of non-European ancestry, defined as further than 10 standard deviations from the European-ancestry population principal components in HapMap 3 (CEU, people of Northern and Western European ancestry living in Utah; TSI, Tuscans in Italy), were excluded from analysis to ensure an ancestrally homogeneous group of samples for association testing. Samples with an inbreeding coefficient >3 standard deviations from the mean of the distribution were excluded, as were unexpectedly related samples. Genotypes available from genotyping on the Illumina Omni 2.5 M array were compared to sequencing genotypes, and samples with < 95% concordance were dropped from the analysis.

For variant quality control, variants with missingness >5% were removed, as were variants out of Hardy-Weinberg equilibrium in controls (*p* < 1 × 10^−6^). Differential missingness between cases and controls was checked and variants with *p* < 1 × 10^−6^ were removed. Variants with extreme low or extreme high depth of coverage (> 6 standard deviations from the mean of the total depth distribution) were also excluded. Finally, the mitochondrial, X and Y chromosomes were excluded from analysis (but will be included in later analyses as sample sizes in Project MinE continue to grow). Approximately 10 million sites were lost during variant quality control.

For identity-by-descent analysis, all non-singleton variants were phased using SHAPEIT2^54^. Subsequently BEAGLE 4.0^55^ was used to detect likely runs of identity by descent between individuals. The hg19 recombination map obtained from the 1000 Genomes Project was used to transform genetic positions from basepairs to centimorgans (cM). Presumed identity by descent segments shorter than one cM were excluded and regions with excessive identity by descent were excluded after visual inspection.

### Structural variation calling and quality control

To calculate the number of structural variation types in each gene, an in-house pipeline was used to filter the variants according to quality score, size, and type of structural variant. An in-house coverage analysis determined that 92% of the desired regions were covered by at least 5 reads. Variants called with 10–20 reads were flagged to be visually inspected to remove false positives. Furthermore, we excluded variants with poor genotyping quality, defined as variants with sequencing quality score less than 20 (out of 100) as well as variants with minimal read depth (less than 5X).

In the pipeline we limited counting of the structural variation to one variant/position to avoid counting the same variants multiple times.

Manta cannot detect small variants, dispersed duplications and gene expansion variants of a reference tandem repeat such as *C9orf72* and *ATXN2*, as the power to assemble variants to break-end resolution falls to zero as break-end repeat length approaches the read size. Furthermore, the power to detect any break-end falls to almost zero as the break-end repeat length approaches the fragment size. Therefore, we used Expansion Hunter and data obtained from real time PCR to confirm *C9orf72* expansion status. Following Expansion Hunter tool instructions, the genome coordinates that were used to confirm *C9orf72* expansion status were chr9:27573527-27573544 and the motif GGCCCC^50^. Additionally, 29 off-target regions were also included to determine the *C9orf72* repeat size. (Please refer to the Expansion Hunter Github page (https://github.com/Illumina/ExpansionHunter) for the exact coordinates of the 29 off-target regions). If 30 or more repeats was reported, an allele was considered expanded^50^.

Furthermore, Manta is unable to detect inversions less than about 200 bases in size. The actual limiting size was not tested; thus, we used the size 200 bp as the threshold in the in-house pipeline and called inverted variants bigger than 200 bp. Manta also cannot detect fully assembled large insertions. Thus, the pipeline included a cut-off limit of 100,000 bp as the tool was not tested beyond this size. As the exact coordinates of inversions and insertions can differ between people, sequence overlap was required for the coding sequence to be counted.

A random selection of BAM files from 30 sequences was manually inspected to ensure that VCF calls of structural variation had corresponding raw source file changes between the BAM and VCF files. A few representative IGV screenshots of the SVs are included in supplementary appendix (Supplementary Data Fig. 1).

### Reporting summary

Further information on research design is available in the Nature Research Reporting Summary linked to this article.

## Supplementary information

Supplementary Information

Reporting Summary

## Data Availability

The data sets used that support the findings in this study are available from The Project MinE consortium public repository. To gain access to the data, an account request must be made to info@projectmine.com. Data access will require the completion of a data access request. Further information about data access can be found at https://www.projectmine.com/research/data-sharing/

## Code availability

We have integrated the code as part of ALSgeneScanner^56^ a tool we developed for analysis of ALS known genes https://github.com/KHP-Informatics/ALSgeneScanner.
