# Supplementary material for: Structural variation analysis of 6,500 whole genome sequences in amyotrophic lateral sclerosis
Source: NPJ Genom Med. 2022 Jan 28;7:8. doi: 10.1038/s41525-021-00267-9 (PMC8799638; doi:10.1038/s41525-021-00267-9)
Supplement: Supplementary file 1 — Supplementary Information [file 41525_2021_267_MOESM1_ESM.pdf]

Structural variation analysis of 6,500 whole genome sequences in amyotrophic lateral sclerosis

Ahmad Al Khleifat[1], Alfredo Iacoangeli[1,2], Joke J.F.A. van Vugt[3], Harry Bowles[1], Matthieu Moisse[4], Ramona A. J. Zwamborn[3], Rick A.A. van der Spek[3], Aleksey Shatunov[1], Johnathan Cooper-Knock[5], Simon Topp[1], Ross Byrne[6], Cinzia Gellera[7], Victoria López[7], Ashley R Jones[1], Sarah Opie-Martin[1], Atay Vural[8], Yolanda Campos[9], Wouter van Rheenen[3], Brendan Kenna[3], Kristel R. Van Eijk[3], Kevin Kenna [3], Markus Weber[10], Bradley Smith[1], Isabella Fogh[1], Vincenzo Silani[7], Karen E Morrison[11], Richard Dobson[2,12], Michael A. van Es[3], Russell L. McLaughlin[6], Patrick Vourc'h[13] , Adriano Chio[14,15], Philippe Corcia[13,16], Mamede de Carvalho[17], Marc Gotkine[18], Monica P. Panades[19], Jesus S. Mora[20], Pamela J. Shaw[5], John E. Landers[21], Jonathan D. Glass[22], Christopher E Shaw[1,23], Nazli Basak[8], Orla Hardiman[23,25], Wim Robberecht[4,26], Philip Van Damme[4,26], Leonard H. van den Berg[3], Jan H. Veldink[3], Ammar Al-Chalabi[1,23].

1. King's College London, Maurice Wohl Clinical Neuroscience Institute, Department of Basic and Clinical Neuroscience, De Crespigny Park, London, SE5 9RX, UK

2. Department of Biostatistics and Health Informatics, Institute of Psychiatry, Psychology and Neuroscience, King's College London, London, U.K.

3. Department of Neurology, UMC Utrecht Brain Center, Utrecht University, The Netherlands

4. KU Leuven – University of Leuven, Department of Neurosciences, Experimental Neurology; VIB Center for Brain & Disease Research, Laboratory of Neurobiology, Leuven, Belgium

5.Sheffield Institute for Translational Neuroscience (SITraN), University of Sheffield, Sheffield, UK

6. Complex Trait Genomics Laboratory, Smurfit Institute of Genetics, Trinity College Dublin, Ireland

7. Department of Neurology and Laboratory of Neuroscience, IRCCS Istituto Auxologico Italiano and Department of Pathophysiology and Transplantation, "Dino Ferrari" Center, Università degli Studi di Milano, Milano, Italy

8.Genomize Inc. Bogazici University, Technology Transfer Region, ETAB, Istanbul, Turkey

9.Mitochondrial pathology Unit, Instituto de Salud Carlos III, Madrid, Spain

10.Neuromuscular Diseases Unit/ALS Clinic, Kantonsspital St. Gallen, 9007, St. Gallen, Switzerland.

11. Faculty of Medicine, Health and Life Sciences, Queen's University Belfast, Belfast, Northern Ireland, UK

12. Institute of Health Informatics, University College London, London, U.K.

13. Centre SLA, CHRU de Tours, Tours, France

14. Rita Levi Montalcini, Department of Neuroscience, ALS Centre, University of Torino, Turin, Italy

15. Azienda Ospedaliera Citta della Salute e della Scienza, Torino, Italy

16. Federation des Centres SLA Tours and Limoges, LITORALS, Tours, France

17. Physiology Institute, Faculty of Medicine, Instituto de Medicina Molecular, University of Lisbon, Lisbon, Portugal

18. Hadassah University Hospital, Jerusalem, Israel

19. Neurology Department, Hospital Universitari de Bellvitge, Barcelona, Spain

20. Hospital San Rafael, Madrid, Spain

21. Department of Neurology, University of Massachusetts Medical School, Worcester, MA, USA

22. Department of Neurology, Center for Neurodegenerative Diseases, Emory University, Atlanta, Georgia USA

23. King's College Hospital, Denmark Hill, London, SE5 9RS, UK

24. Academic Unit of Neurology, Trinity College Dublin, Trinity Biomedical Sciences Institute, Dublin, Republic of Ireland

25. Department of Neurology, Beaumont Hospital, Dublin, Republic of Ireland

26.Neurology Department, University Hospitals Leuven, Leuven, Belgium

Correspondence: Ammar Al-Chalabi, Department of Basic and Clinical Neuroscience, Maurice Wohl Clinical Neuroscience Institute, 5 Cutcombe Road, London SE5 9RX, UK. Tel: +44 2078485192, +44 2078485190. E-mail: ammar.al-chalabi@kcl.ac.uk

Supplementary Tables

Supplementary Table 1. List of 25 ALS genes identified by literature review.

**Gene**	**ALS Locus Number**	**Key Reference**
ALS2		10.1038/ng1001-160.
ANG	ALS 9	10.1038/srep41996.
ATXN2	ALS 13	10.1038/nature09320
C9orf72	ALS-FTD1	10.1016/j.neuron.2011.09.011.
CHCHD10		10.1093/brain/awu138
DAO	ALS	10.1073/pnas.0914128107
FUS	ALS 6	10.1126/science.1165942
HNRNPA1	ALS 20	10.1038/nature11922
MOBP		10.1038/ng.3622
NEK1	ALS 24	10.1038/ng.3626
OPTN	ALS 12	10.1126/science.aaa3650
PFN1	ALS 18	10.1038/nature11280
SCFD1		10.1038/ng.3622
SOD1	ALS 1	10.1038/362059a0
SPG11	ALS 5	10.1126/science.aaa3650
SQSTM1		10.1001/archneurol.2011.250
SETX	ALS 4	10.1086/421054
TARDBP	ALS 10	10.1126/science.1154584
TBK1		10.1038/ng.3622
TUBA4A	ALS 22	10.1016/j.neuron.2014.09.027
UBQLN2	ALS 15	10.1038/nature10353
UNC13A		10.1038/ng.3622
VAPB	ALS 8	10.1086/425287
VCP	ALS 14	10.1016/j.neuron.2010.11.036

The literature review was performed using several databases, including PubMed, MEDLINE, and EMBASE, to identify all articles reporting the contribution of genetic variations to the development of the disease or the modification of the phenotype in ALS.

Supplementary Table 2. Structural variation in sporadic ALS.

VCP **Inversion**	**Cases (freq)**	**Controls (freq)**
**Present**	2430 (0·56)	669 (0·35)
**Absent**	1885 (0·43)	1211 (0·64)
**Total**	4315	1880
C9orf72 **Expansion**	**Cases (freq)**	**Controls (freq)**
**Present**	244 (0·05)	4 (0·002)
**Absent**	4071 (0·94)	1876 (0·98)
**Total**	4315	1880
ERBB4 **Insertion**	**Cases (freq)**	**Controls (freq)**
**Present**	2001 (0·46)	476 (0·25)
**Absent**	2314 (0·53)	1404 (0·74)
**Total**	4315	1880

There were three genes in which structural variation was associated with ALS: C9orf72, VCP, and ERBB4. SV = structural variation; freq = frequency.

Supplementary Table 3. Assessment of deletion variation burden between people with ALS and healthy controls.

Gene DEL	Estimate (%)	SE of estimate (%)	p-Value
ALS2	-0.008	0.032	0.794
ANG	0.095	0.232	0.681
ATXN2	-0.006	0.017	0.706
CHCHD10	NA	NA	NA
DAO	0.028	0.031	0.373
ERBB4	-0.001	0.004	0.720
FUS	NA	NA	NA
HNRNPA1	NA	NA	NA
MOBP	0.022	0.008	0.009
NEK1	0.032	0.038	0.390
OPTN	-0.028	0.015	0.064
PFN1	0.307	0.466	0.510
SCFD1	-0.012	0.052	0.813
SETX	-0.020	0.022	0.367
SOD1	0.158	0.129	0.220
SPG11	-0.031	0.047	0.512
SQTM1	-0.056	0.049	0.252
TARDBP	0.000	0.064	1.000
TBK1	-0.006	0.056	0.918
TUBA4A	0.146	0.190	0.442
UBQLN2	NA	NA	NA
UNC13A	0.010	0.010	0.288
VAPB	-0.007	0.012	0.560
VCP	-0.242	0.327	0.560

Assessment of deletion variation burden between people with ALS and healthy controls using a linear mixed model in 25 known ALS genes. The effect of structural variation on ALS risk in each gene was examined independently and assessed using multivariable linear regression after correcting for different sequencing platforms. Population stratification, principal components of ancestry, centre, age and sex were added as covariates to the model. Estimate indicate the difference in mean burden between the groups. DEL =deletion.

Supplementary Table 4. Assessment of insertion variation burden between people with ALS and healthy controls.

Gene- INS	Estimate (%)	SE of estimate (%)	p-Value
ALS2	0.005	0.017	0.755
ANG	NA	NA	NA
ATXN2	0.089	0.055	0.108
CHCHD10	NA	NA	NA
DAO	-0.103	0.077	0.185
ERBB4	0.024	0.008	0.0003
FUS	NA	NA	NA
HNRNPA1	NA	NA	NA
MOBP	NA	NA	NA
NEK1	0.044	0.065	0.498
OPTN	-0.034	0.109	0.757
PFN1	NA	NA	NA
SCFD1	0.027	0.043	0.531
SETX	0.091	0.231	0.694
SOD1	0.088	0.232	0.703
SPG11	0.324	0.327	0.322
SQTM1	NA	NA	NA
TARDBP	0.000	0.267	0.999
TBK1	NA	NA	NA
TUBA4A	-0.151	0.327	0.644
UBQLN2	NA	NA	NA
UNC13A	0.047	0.026	0.074
VAPB	-0.026	0.011	0.013
VCP	NA	NA	NA

Supplementary Table 4. Assessment of insertion variation burden between people with ALS and healthy controls using a linear mixed model in 25 known ALS genes. The effect of structural variation on ALS risk in each gene was examined independently and assessed using multivariable linear regression after correcting for different sequencing platforms. Population stratification, principal components of ancestry, centre, age and sex were added as covariates to the model. Estimate indicate the difference in mean burden between the groups. INS = insertion.

Supplementary Table 5. Assessment of inversion variation burden between people with ALS and healthy controls.

Gene- INV	Estimate (%)	SE of estimate (%)	p-Value
ALS2	NA	NA	NA
ANG	NA	NA	NA
ATXN2	-0.035	0.017	0.033
CHCHD10	-0.010	0.010	0.288
DAO	0.374	0.327	0.253
ERBB4	0.107	0.101	0.288
FUS	NA	NA	NA
HNRNPA1	NA	NA	NA
MOBP	0.007	0.019	0.703
NEK1	0.315	0.326	0.334
OPTN	-0.686	0.461	0.136
PFN1	-0.217	0.093	0.019
SCFD1	-0.667	0.461	0.148
SETX	0.333	0.461	0.469
SOD1	NA	NA	NA
SPG11	-0.008	0.206	0.967
SQTM1	0.013	0.009	0.148
TARDBP	0.203	0.188	0.280
TBK1	NA	NA	NA
TUBA4A	0.167	0.231	0.469
UBQLN2	0.154	0.154	0.505
UNC13A	-0.039	0.019	0.044
VAPB	0.304	0.326	0.351
VCP	0.030	0.007	0.0002

Assessment of inversion variation burden between people with ALS and healthy controls using a linear mixed model in 25 known ALS genes. The effect of structural variation on ALS risk in each gene was examined independently and assessed using multivariable linear regression after correcting for different sequencing platforms. Population stratification, principal components of ancestry, centre, age and sex were added as covariates to the model. Estimate indicate the difference in mean burden between the groups. INV = inversion.

Supplementary Table 6. Assessment of duplications variation burden between people with ALS and healthy controls.

Gene-DUP	Estimate (%)	SE of estimate (%)	p-Value
ALS2	NA	NA	NA
ANG	NA	NA	NA
ATXN2	-0.024	0.154	0.875
CHCHD10	NA	NA	NA
DAO	NA	NA	NA
ERBB4	NA	NA	NA
FUS	NA	NA	NA
HNRNPA1	NA	NA	NA
MOBP	NA	NA	NA
NEK1	NA	NA	NA
OPTN	0.308	0.462	0.505
PFN1	NA	NA	NA
SCFD1	-0.192	0.231	0.406
SETX	NA	NA	NA
SOD1	0.000	0.462	0.505
SPG11	NA	NA	NA
SQTM1	NA	NA	NA
TARDBP	NA	NA	NA
TBK1	NA	NA	NA
TUBA4A	NA	NA	NA
UBQLN2	NA	NA	NA
UNC13A	-0.192	0.327	0.556
VAPB	0.086	0.154	0.578
VCP	NA	NA	NA

Assessment of duplications variation burden between people with ALS and healthy controls using a linear mixed model in 25 known ALS genes. The effect of structural variation on ALS risk in each gene was examined independently and assessed using multivariable linear regression after correcting for different sequencing platforms. Population stratification, principal components of ancestry, centre, age and sex were added as covariates to the model. Estimate indicate the difference in mean burden between the groups.

Supplementary Table 7. Phenotypic information for people included in the survival analysis.

Site of onset	Total number of people	Number of events (death)	Censored
**Bulbar**	1174	1017	157
**Respiratory**	63	54	9
**Spinal**	2780	2204	576
**Overall**	4017	3275	742

Description of phenotypic information for people included in the survival analysis, including the site of onset and number who died during the study period.

Supplementary Figure 1. Snapshots of IGV browser.

***ERBB4* - gene insertion**

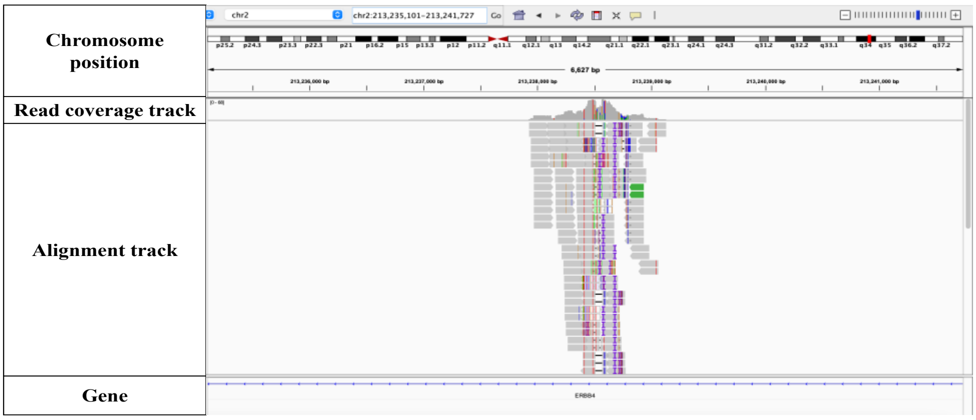

***ERBB4* - gene insertion**

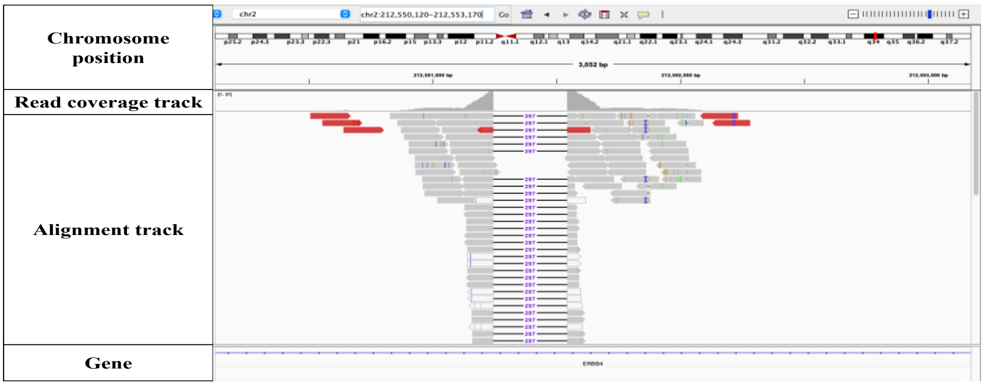

***ERBB4* - gene insertion**

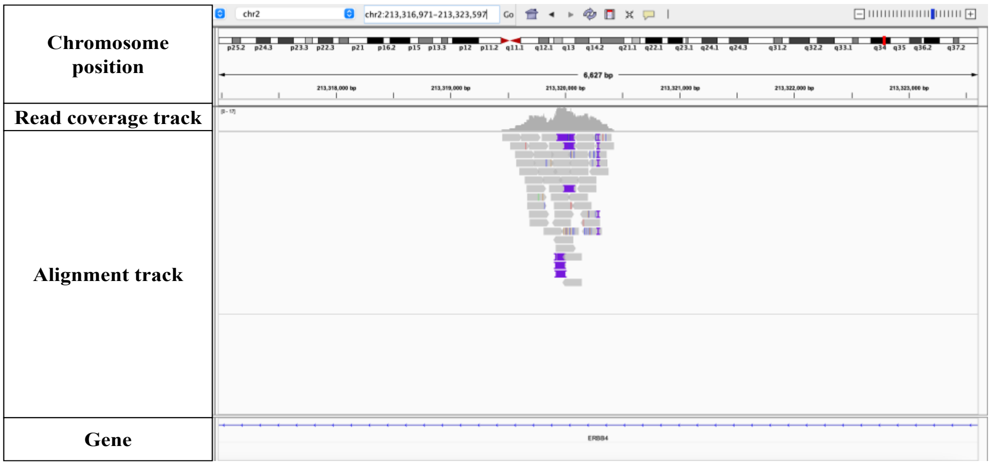

Snapshots of IGV browser. In a gapped alignment, IGV indicates insertions with respect to the reference gene ERBB4 with a purple I or red "I" for large insertions.

Survival Curves

Supplementary Figure 2. Cox survival analysis for people with ALS and *C9orf72* gene expansion.

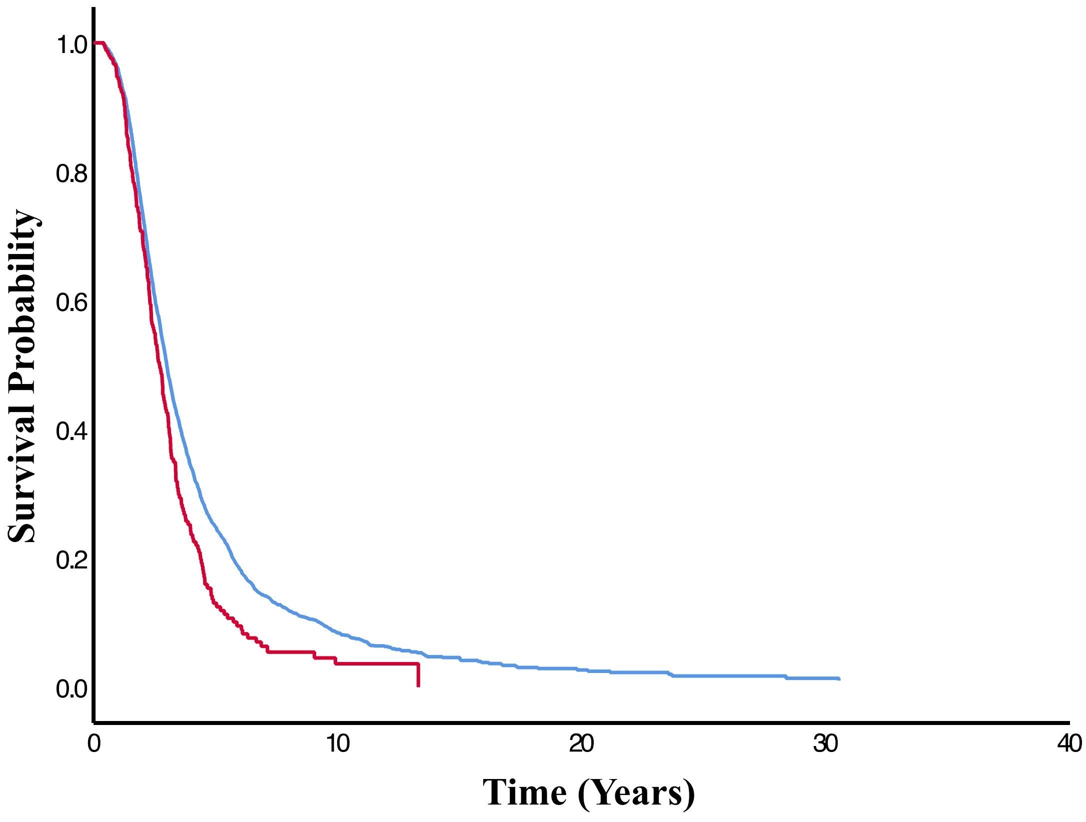

Cox survival analysis showed that people with C9orf72-mediated ALS (red line) had worse survival than people with ALS with no C9orf72 gene expansion (blue line). The curves were significantly different ($p = 3 \times 10^{-6}$). Time (Years) indicates time since symptom onset.

Supplementary Figure 3. Cox survival analysis for people with ALS and *VCP* gene inversion.

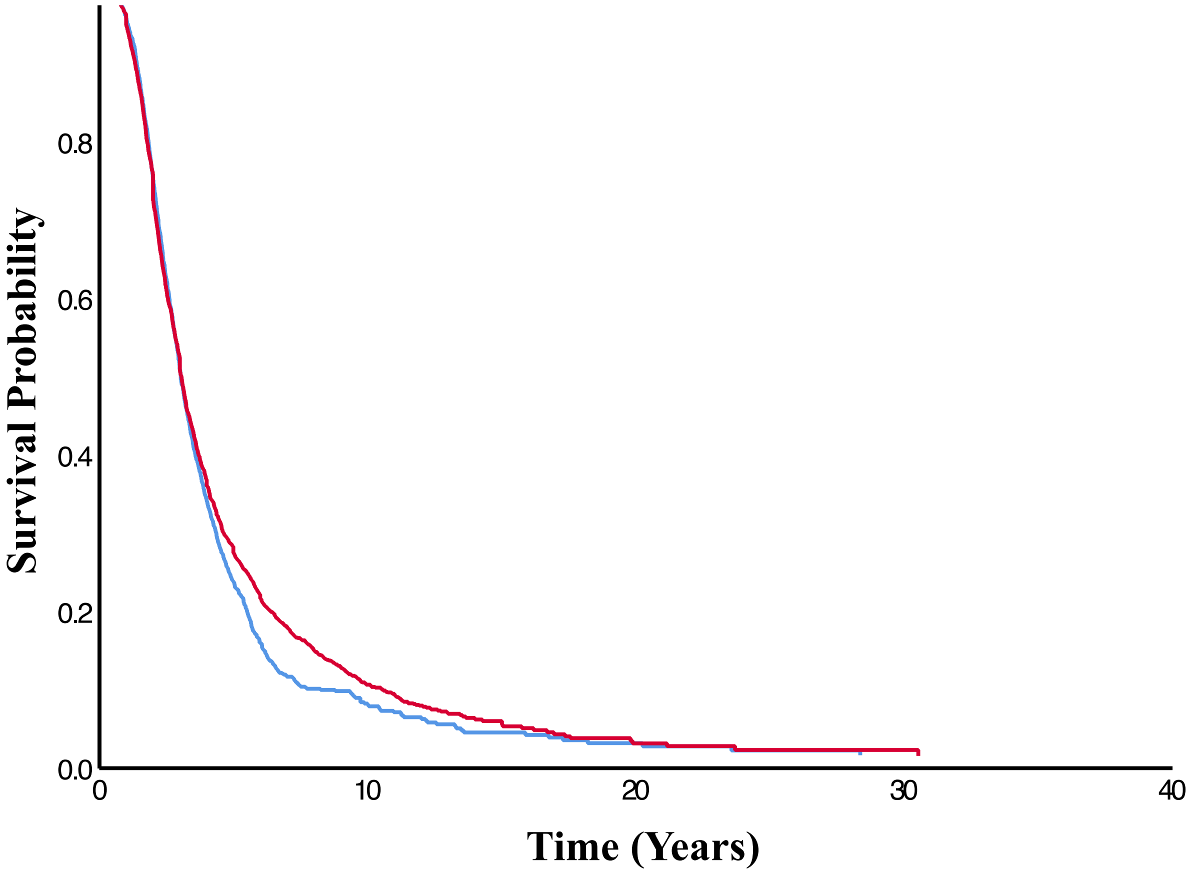

Cox survival analysis showed that people with ALS and VCP gene inversion (red line) had longer survival than those with ALS and no VCP gene inversion (blue line). The curves were significantly different (p = 0·002). Time (Years) indicates time since symptom onset.

Supplementary Figure 4. Cox survival analysis for people with ALS and *ERBB4* gene insertion.

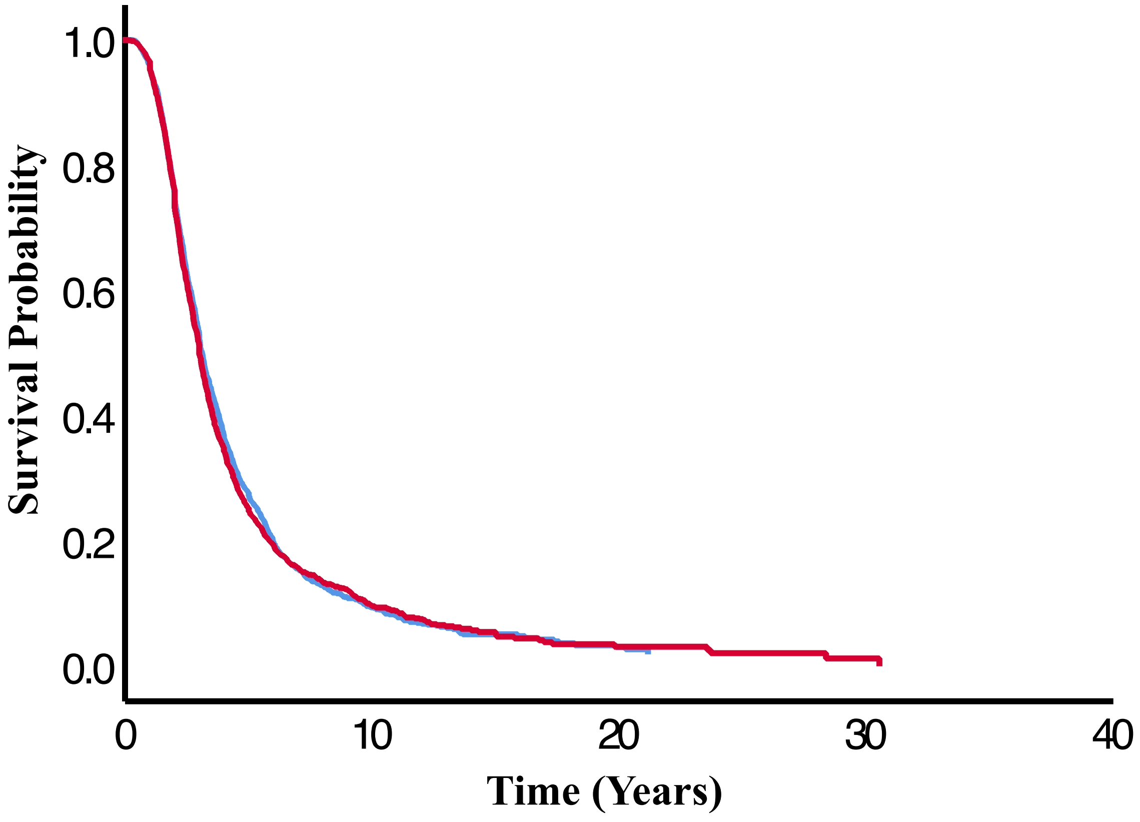

Cox survival analysis showed that there was no difference in survival between people with ALS and ERBB4 gene insertion (red line) and those with ALS and no ERBB4 gene insertion (blue line) (p = 0·09). Time (Years) indicates time since symptom onset.

Supplementary Figure 5. Kaplan–Meier survival curves of respiratory onset amyotrophic lateral sclerosis.

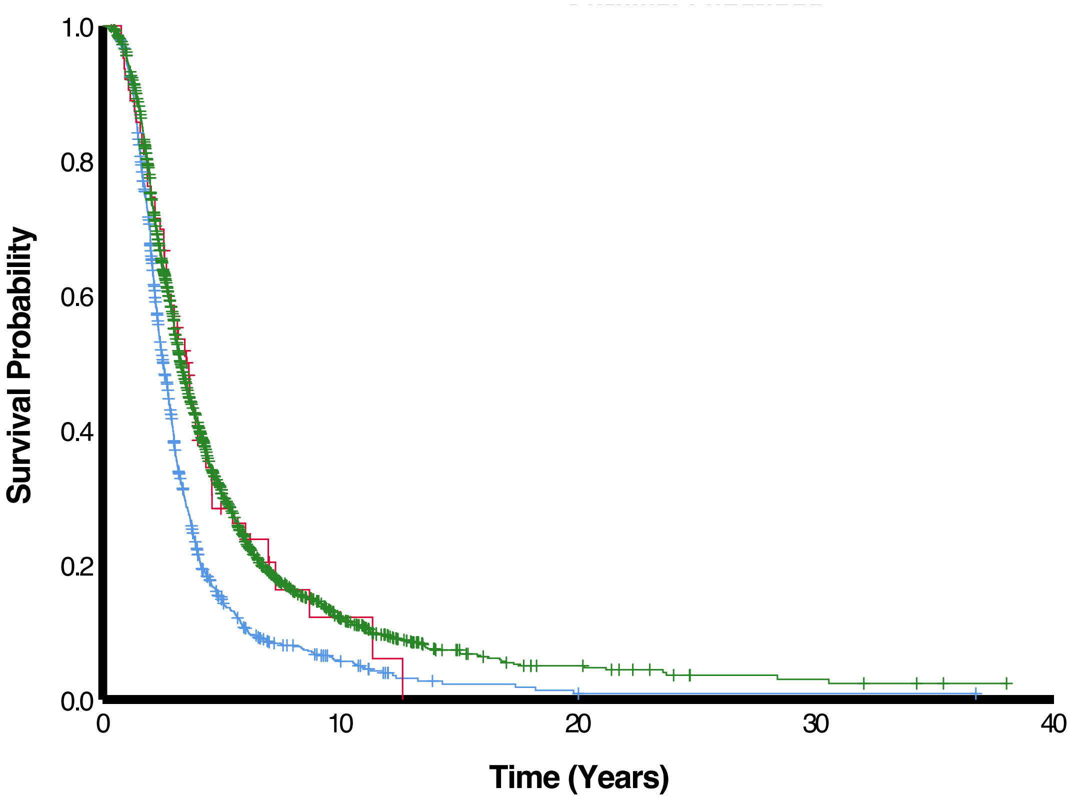

Number at risk	0	10	20	30	40
Bulbar	1174	984	29	4	
Respiratory	63	47	7		
Spinal	2780	2011	175	14	

Kaplan–Meier survival curves of respiratory onset amyotrophic lateral sclerosis (red line) compared with bulbar onset ALS (blue line) and spinal onset ALS (green line). Censored individuals are indicated by a cross sign. Time (Years) indicates time since symptom onset. Overall, 742 were censored (48·38%), of which 157 (13·40%) had bulbar onset, 9 (14·28%) had respiratory onset and 576 (20·70%) had spinal onset ALS. The curves were significantly different ($p=6{\cdot}6x10^{-34}$, log rank).

Supplementary Figure 6. Kaplan–Meier survival curves of respiratory onset ALS with no *ERRB4* insertion.

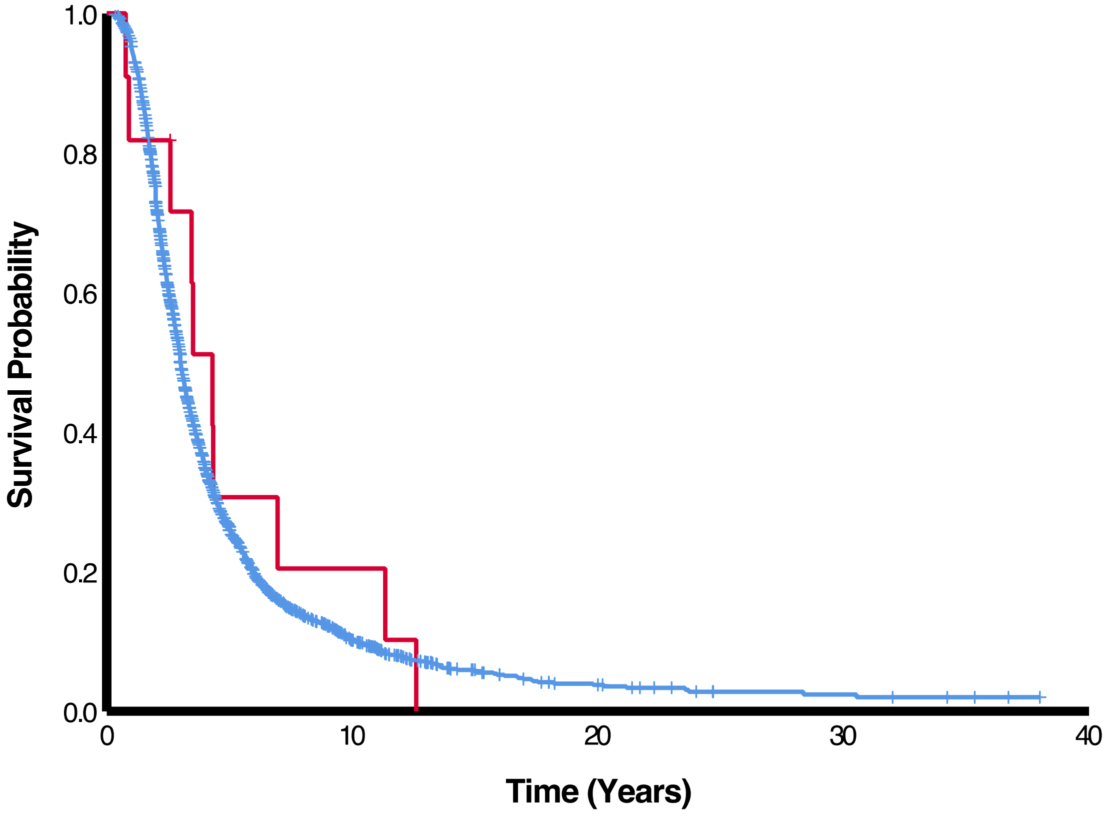

Kaplan–Meier survival curves of respiratory onset ALS with no ERRB4 insertion (blue line) compared with respiratory onset ALS with ERRB4 insertion (red line). Censored individuals are indicated by cross sign. The curves were not significantly different (p = 0·15, log rank). Time (Years) indicates time since symptom onset.

Supplementary Figure 7. Cox survival analysis for people with ALS and combined *C9orf72* gene expansion, *VCP* gene inversion, and *ERBB4* gene insertion.

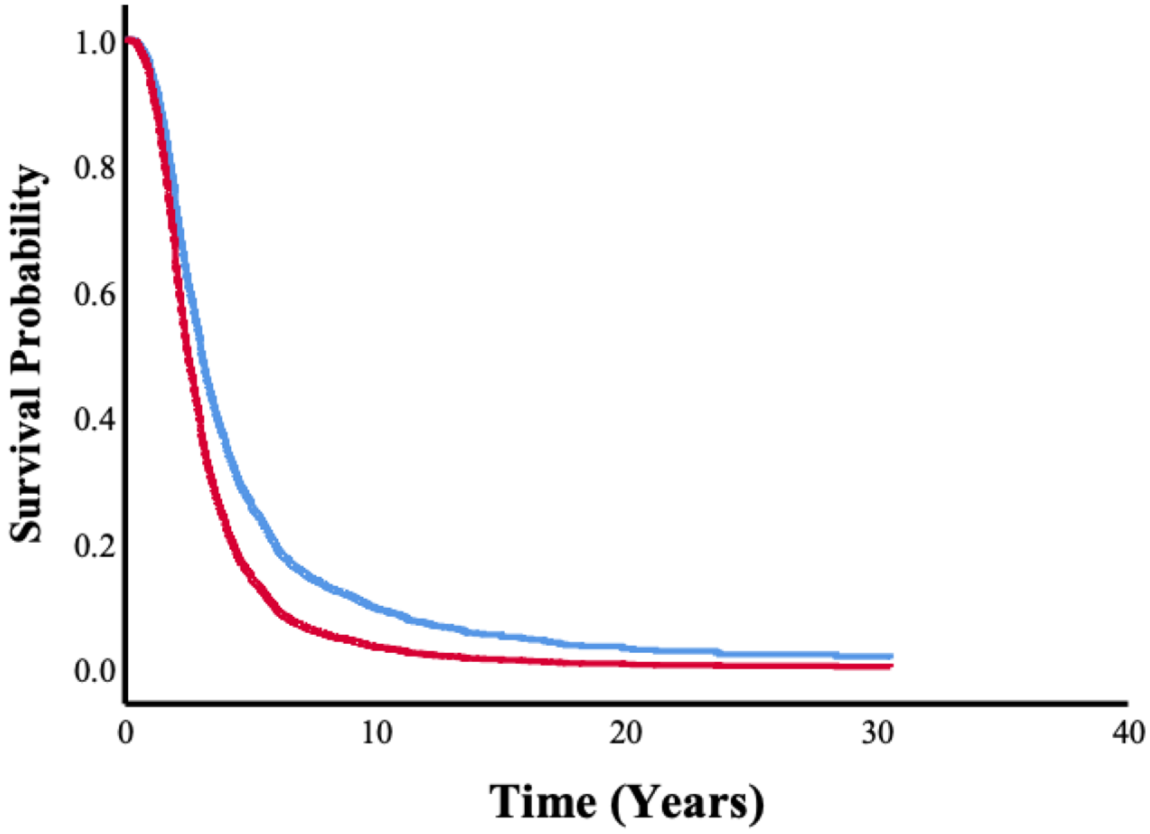

Cox survival analysis showed that people with ALS and people with ALS carrying three variations simultaneously: C9orf72 expansion, VCP inversion and ERBB4 insertion (red line) had worse survival (p = 6.7 x 10^{-5}*) than people with ALS with no overlapping structural variations in C9orf72, VCP, and ERBB4 genes (blue line).*
