# Supplementary material for: Structural variation analysis of 6,500 whole genome sequences in amyotrophic lateral sclerosis
Source: NPJ Genom Med. 2022 Jan 28;7:8. doi: 10.1038/s41525-021-00267-9 (PMC8799638; doi:10.1038/s41525-021-00267-9)
Supplement: Supplementary file 2 — Reporting Summary [file 41525_2021_267_MOESM2_ESM.pdf]

nature research

Corresponding author(s): DBRP. npj Genomic Medicine
NPJGENMED-00756-T Major Revision
Professor Ammar Al-Chalabi

Last updated by author(s): Sep 24, 2021

Reporting Summary

Nature Research wishes to improve the reproducibility of the work that we publish. This form provides structure for consistency and transparency in reporting. For further information on Nature Research policies, see our Editorial Policies and the Editorial Policy Checklist.

Statistics

For all statistical analyses, confirm that the following items are present in the figure legend, table legend, main text, or Methods section.

n/a	Confirmed	
☐	☒	The exact sample size (*n*) for each experimental group/condition, given as a discrete number and unit of measurement
☐	☒	A statement on whether measurements were taken from distinct samples or whether the same sample was measured repeatedly
☐	☒	The statistical test(s) used AND whether they are one- or two-sided<br>*Only common tests should be described solely by name; describe more complex techniques in the Methods section.*
☐	☒	A description of all covariates tested
☐	☒	A description of any assumptions or corrections, such as tests of normality and adjustment for multiple comparisons
☐	☒	A full description of the statistical parameters including central tendency (e.g. means) or other basic estimates (e.g. regression coefficient) AND variation (e.g. standard deviation) or associated estimates of uncertainty (e.g. confidence intervals)
☐	☒	For null hypothesis testing, the test statistic (e.g. *F*, *t*, *r*) with confidence intervals, effect sizes, degrees of freedom and *P* value noted<br>*Give P values as exact values whenever suitable.*
☒	☐	For Bayesian analysis, information on the choice of priors and Markov chain Monte Carlo settings
☒	☐	For hierarchical and complex designs, identification of the appropriate level for tests and full reporting of outcomes
☐	☒	Estimates of effect sizes (e.g. Cohen's *d*, Pearson's *r*), indicating how they were calculated

Our web collection on statistics for biologists contains articles on many of the points above.

Software and code

Policy information about availability of computer code

Data collection	Clinical information including sex, age at first symptoms, age at onset, site of onset, survival status, and disease duration, was obtained from the patient record according to standard definitions as defined by the SOPHIA standard operating procedures.<br><br>Whole-genome sequencing<br>DNA was isolated from venous blood using standard methods. DNA concentration was set at 100ng/ul as measured by fluorimeter with the PicoGreen® dsDNA quantitation assay. DNA integrity was assessed using gel electrophoresis. All samples were sequenced using Illumina's FastTrack services (San Diego, CA, USA) on the Illumina HiSeq 2000 (100bp paired-end reads) and HiSeqX platforms (150bp paired end reads) (20), using PCR-free library preparations. Binary sequence alignment/map formats (BAM) were generated for each individual. The Project MinE genomes were aligned with Isaac (Illumina) to hg19. The details of the Isaac alignment and variant calling pipelines are discussed in Project MinE design 18 and Isaac protocol.
Data analysis	We have integrated the code as part of ALSgeneScanner a tool we developed for analysis of ALS known genes https://github.com/KHP-Informatics/ALSgeneScanner .

For manuscripts utilizing custom algorithms or software that are central to the research but not yet described in published literature, software must be made available to editors and reviewers. We strongly encourage code deposition in a community repository (e.g. GitHub). See the Nature Research guidelines for submitting code & software for further information.

Data

Policy information about availability of data

All manuscripts must include a data availability statement. This statement should provide the following information, where applicable:

- Accession codes, unique identifiers, or web links for publicly available datasets
- A list of figures that have associated raw data
- A description of any restrictions on data availability

The data sets and code used that support the findings in this study are available from the corresponding author upon reasonable request. We have integrated the code as part of ALSgeneScanner a tool we developed for analysis of ALS known genes https://github.com/KHP-Informatics/ALSgeneScanner .

Field-specific reporting

Please select the one below that is the best fit for your research. If you are not sure, read the appropriate sections before making your selection.

☒ Life sciences ☐ Behavioural & social sciences ☐ Ecological, evolutionary & environmental sciences

For a reference copy of the document with all sections, see nature.com/documents/nr-reporting-summary-flat.pdf

Life sciences study design

All studies must disclose on these points even when the disclosure is negative.

Sample size

There were 6,580 whole genome sequences, reducing to 6,195 samples (4,315 from people with ALS and 1,880 controls) after quality control, with minimum ~25x coverage across each sample. Of those with ALS, 4,236 had apparently sporadic ALS and 79 had familial ALS. The male-female ratio was 2:1. There were 4,287 people sequenced using the HiSeqX Illumina platform, and 1,908 sequenced using the HiSeq2000 platform. This is the largest WGS data available for ALS therefore we have sufficient power to preform the SV analysis done in this manuscript.

Data exclusions

There were 6,580 whole genome sequences, reducing to 6,195 samples (4,315 from people with ALS and 1,880 controls) after quality control. Quality control was performed separately on the genotyped data of each population according to Project MinE methods published previously22 (Supplementary appendix).

Replication

We have tested the main SVs using a two tools, Manta and Pindel, in 200 randomly selected samples and find excellent agreement with our initial analysis. Furthermore, random selection of BAM files from 30 sequences were manually inspected to ensure that variant call format (VCF) calls of structural variation had corresponding raw source file changes. Raw source file changes refer to a comparison between the BAM and VCF files to confirm the two correspond. Few representative IGV screenshots of the SVs are included can be found in the supplementary appendix (Supplementary Figure 1).

Randomization

cases and controls are similarly distributed between the platforms (HiSeq2500 66% cases, 34% controls and HiSeqX 70% cases and 30% controls) (p = 0.54). To overcome this potential weakness, all the samples used were sequenced at the same Illumina lab using two industry-leading sequencing platform for all samples, as well as designing the study to minimize batch effects by having cases and controls sharing the same sequencing plate, and taking sequencing platform into account as a covariate in our analyses.

Blinding

This is a genetic association analysis therefore information about cases and controls is required.

Reporting for specific materials, systems and methods

We require information from authors about some types of materials, experimental systems and methods used in many studies. Here, indicate whether each material, system or method listed is relevant to your study. If you are not sure if a list item applies to your research, read the appropriate section before selecting a response.

Materials & experimental systems

n/a	Involved in the study
☒	☐ Antibodies
☒	☐ Eukaryotic cell lines
☒	☐ Palaeontology and archaeology
☒	☐ Animals and other organisms
☐	☒ Human research participants
☒	☐ Clinical data
☒	☐ Dual use research of concern

Methods

n/a	Involved in the study
☒	☐ ChIP-seq
☒	☐ Flow cytometry
☒	☐ MRI-based neuroimaging

Human research participants

Policy information about studies involving human research participants

Population characteristics	There were 6,580 whole genome sequences, reducing to 6,195 samples (4,315 from people with ALS and 1,880 controls) after quality control, with minimum ~25x coverage across each sample. Of those with ALS, 4,236 had apparently sporadic ALS and 79 had familial ALS. The male-female ratio was 2:1. Overall, 31 had cognitive impairment, 20 had ALS-frontotemporal dementia (ALS-FTD) and 63 had respiratory onset ALS. There were 4,287 people sequenced using the HiSeqX Illumina platform, and 1,908 sequenced using the HiSeq2000 platform
Recruitment	Samples were from the international Project MinE whole genome sequencing consortium and derived from seven countries: the USA, Ireland, Belgium, the Netherlands, Spain, Turkey, and the United Kingdom.
Ethics oversight	Informed consent for genetic research was obtained from all participants, approved by the Trent Research Ethics Committee 08/H0405/60.

Note that full information on the approval of the study protocol must also be provided in the manuscript.
